# Influence of bulky yet flexible *N*-heterocyclic carbene ligands in gold catalysis

**DOI:** 10.3762/bjoc.11.196

**Published:** 2015-10-02

**Authors:** Alba Collado, Scott R Patrick, Danila Gasperini, Sebastien Meiries, Steven P Nolan

**Affiliations:** 1 EaStCHEM School of Chemistry, University of St Andrews, St Andrews, KY16 9ST, UK; 2 Chemistry Department, College of Science, King Saud University, Riyadh 11451, Saudi Arabia

**Keywords:** catalysis, flexible and bulky ligands, gold, ligand design, N-heterocyclic carbenes

## Abstract

Three new Au(I) complexes of the formula [Au(NHC)(NTf_2_)] (NHC = *N*-heterocyclic carbene) bearing bulky and flexible ligands have been synthesised. The ligands studied are IPent, IHept and INon which belong to the ‘ITent’ (‘Tent’ for ‘tentacular’) family of NHC derivatives. The effect of these ligands in gold-promoted transformations has been investigated.

## Introduction

**Figure 1:**
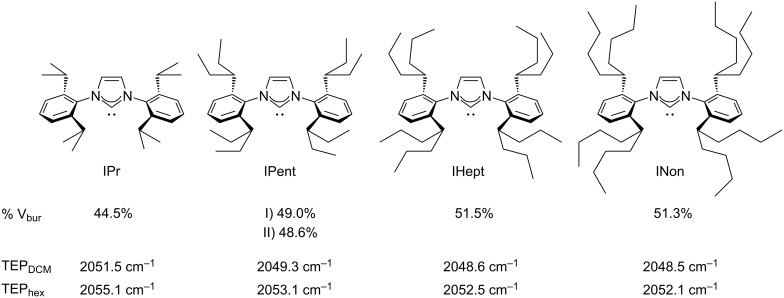
‘ITent’ family of ligands, including IPr. First row: percentage buried volume (% V_bur_) calculated in [Au(ITent)Cl] complexes using the Samb*V*ca software [42–45]. Second and third row: Tolman Electronic Parameter (TEP) calculated from IR measurements in solution of the [Ni(ITent)CO_3_] complexes [21,42].

Homogeneous gold catalysis has witnessed an exponential growth in the last 15 years [1–12]. Gold complexes have been shown to be efficient catalysts in a wide variety of transformations [1–12]. *N*-heterocyclic carbenes (NHC) have attracted particular attention as ancillary ligands due to their donating properties and steric hindrance [13–20]. These can be easily tuned by modifying the architecture of the imidazole ring, typically, by changing the *N*-substituents or the backbone [13–20]. One of our research interests is the study of the electronic and steric properties of new *N*-heterocyclic carbenes and their influence in catalysis upon coordination to a metal centre. We have recently reported the synthesis of the ‘ITent’ family (‘Tent’ stands for ‘tentacular’) of NHC carbenes [21] which comprises IPent, first utilised by Organ, IHept, and INon (Figure 1). IPr [22], which is one of the most commonly used NHC derivatives, can be considered as the simplest congener of the ITent family (Figure 1). These ligands belong to the class of NHC with ‘flexible sterics’, i.e., ligands capable of adjusting their steric hindrance towards incoming substrates and, at the same time, stabilising low-valent species. This concept, first proposed by Glorius [23–26], has been applied by a number of research groups to metal-catalysed transformations, leading to improvements in catalytic activity over other known systems [27–41]. The ITent ligands have been successfully used in challenging Pd cross-coupling reactions and other Pd-promoted transformations [21–41].

The electronic and steric properties of the ITent family have been determined [21,42,45] and compared to the parent ligand IPr. The observed trend for both electronic and steric properties is as follows: IPr << IPent < IHept ≈ INon showing that an increase in the length of the chain translates into an increase of the donating properties and the steric hindrance. The limit of this increment was found to lie between IHept and INon, where the additional carbon atoms did not have a significant impact on the properties of the ligands.

Herein we report the catalytic activity of gold complexes containing the ITent ligands.

## Results and Discussion

### Synthesis of complexes

**Scheme 1:**
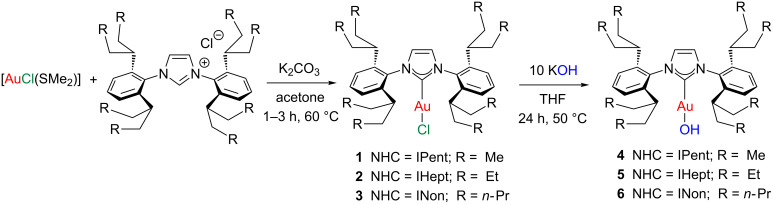
Synthesis of gold complexes bearing the ITent ligands.

We have recently reported the synthesis of [Au(ITent)Cl] and [Au(ITent)(OH)] derivatives following the synthetic protocol shown in Scheme 1 [45].

**Scheme 2:**
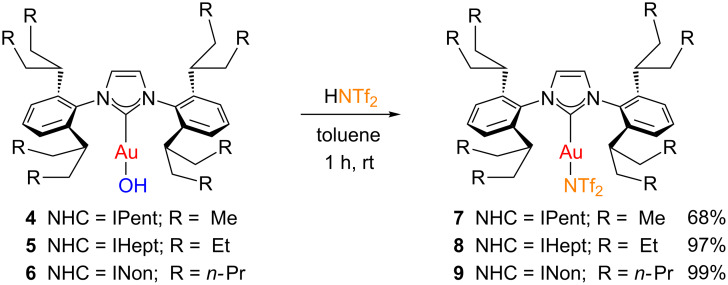
Silver-free synthesis of [Au(ITent)(NTf_2_)] complexes.

In order to study the impact of the ITent ligands in gold catalysis, we sought to synthesise the corresponding Gagosz-type derivatives [46–47]. These complexes, bearing a labile NTf_2_ group, do not require additives to promote catalytic transformations. Gagosz-type species have been typically prepared by reacting the corresponding [Au(L)Cl] derivative (L = PR_3_, NHC) with AgNTf_2_ [46–47]. Alternative protocols that avoid the use of silver salts involve the treatment of gold-hydroxide [48] or gold-acetonyl [49] complexes with trifluoromethanesulfonimide. Following this silver-free procedure, [Au(ITent)(OH)] complexes **4**–**6** were reacted with HNTf_2_ to obtain the corresponding [Au(ITent)(NTf_2_)] species **7**–**9** which were isolated as white solids in good to excellent yields (Scheme 2).

Complexes **7**–**9** were characterised by ^1^H, ^13^C{^1^H}, and ^19^F{^1^H} NMR spectroscopies and elemental analysis. The ^13^C{^1^H} NMR spectra of all the complexes showed a singlet in the low field region of the spectra corresponding to the carbenic carbon atom of the molecule. These signals appear at 167.5–167.8 ppm, in agreement with other [Au(NHC)(NTf_2_)] complexes bearing unsaturated NHC ligands [46,50]. Each ^19^F{^1^H} NMR spectrum contains a singlet at ca. −76 ppm, in agreement with the presence of an inner-sphere NTf_2_ ligand [50].

### Catalytic transformations

Once fully characterised, the catalytic activity of the new complexes was investigated. To this end, three model reactions were selected: alkyne hydration, nitrile hydration, and the synthesis of homoallylic ketones. To place these results into context, [Au(IPr)(NTf_2_)] (**10**) [46] was also tested under the same conditions.

#### Alkyne hydration

Alkyne hydration is an attractive transformation to generate ketones from alkynes with high atom economy. A number of gold complexes have been shown to be active in this transformation [51–58]. Much effort has been devoted to the development of more sustainable gold-promoted protocols and several advances have been made in this field, e.g., very low catalyst loadings (10–1000 ppm) [51–53,56] have been achieved and the transformation has been successfully performed in aqueous media [54,57]. Due to its relevance and importance, the hydration of phenylacetylene was selected as a model transformation to explore the different activity of complexes **7**–**9**.

The reactions were performed under the reported optimised conditions for this transformation: using a 2:1 mixture of 1,4-dioxane/water at 80 °C [56]. All complexes showed excellent catalytic activity at 0.5 mol % catalyst loading, after 3 hours, and full conversion to the ketone was observed in all cases (Table 1, entries 1–4). In order to determine the influence of the ligands in the catalytic activity, the catalyst loading was reduced.

**Table 1:** Influence of the ITent ligands in gold-catalysed alkyne hydration.^a^

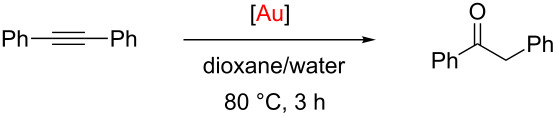				

entry	complex	[Au] (mol %)	time (h)	conversion (%)^b^

1	[Au(IPr)(NTf_2_)] (**10**)	0.5	3	>99
2	[Au(IPent)(NTf_2_)] (**7**)	0.5	3	>99
3	[Au(IHept)(NTf_2_)] (**8**)	0.5	3	>99
4	[Au(INon)(NTf_2_)] (**9**)	0.5	3	>99
5	[Au(IPr)(NTf_2_)] (**10**)	0.25	3	87
6	[Au(IPent)(NTf_2_)] (**7**)	0.25	3	68
7	[Au(IHept)(NTf_2_)] (**8**)	0.25	3	77
8	[Au(INon)(NTf_2_)] (**9**)	0.25	3	70

^a^Reaction conditions: [Au(NHC)(NTf_2_)] (0.5 mol %), phenylacetylene (0.5 mmol), 1,4-dioxane/water (2:1, 1 mL), or [Au(NHC)(NTf_2_)] (0.25 mol %), phenylacetylene (1.0 mmol), 1,4-dioxane/water (2:1, 2 mL). ^b^GC conversion, average of at least two runs.

When the catalyst loading was reduced to 0.25 mol % and the reactions were analysed after 3 h, the differences between the ligands became evident. Good conversions were obtained in all cases (Table 1, entries 5–8). However, the best conversion was observed when complex **10** bearing the IPr ligand was used (Table 1, entry 5). Complexes **7**–**9** afforded lower conversions, with [Au(IHept)(NTf_2_)] (**8**) being the most efficient amongst them (Table 1, entries 6–9).

#### Nitrile hydration

Once it was shown that the use of the ITent ligands in the gold-catalysed hydration of phenylacetylene did not improve upon the performance of the parent IPr ligand, we explored the behaviour of the [Au(ITent)(NTf_2_)] complexes in the hydration of nitriles. Mono- and digold complexes have been shown to be efficient catalysts in this transformation [52,59–60] which proceeds with 100% atom economy. In addition, previous findings showed that the digold complex, [{Au(NHC)}_2_(μ-OH)][BF_4_], bearing IPent was more efficient than the IPr analogue in this reaction [52]. This result encouraged us to test the influence of the ITent ligands in the hydration of nitriles promoted by monogold species.

The reactions were conducted in a 1:1 mixture THF/water and heated at 140 °C under microwave irradiation [60]. Low catalyst loadings were employed in order to observe the differences between the four catalysts studied. At 1 mol %, the complexes showed poor to good catalytic activity depending on the ligand (Table 2). As we observed for the hydration of alkynes, the ITent series were found to be less active than complex **10**, which afforded the desired product in 56% conversion (Table 2, entry 1). However, in this case, the gold complex bearing IPent (**7**) was more efficient than the IHept (**8**) and INon (**9**) derivatives (Table 2, entries 2–4).

**Table 2:** Influence of the ITent ligands in gold-catalysed nitrile hydration.^a^

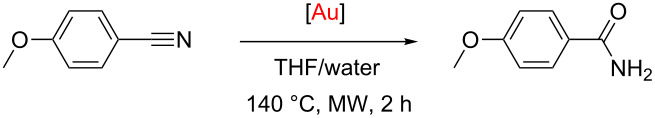				

entry	complex	[Au] (mol %)	time (h)	conversion (%)^b^

1	[Au(IPr)(NTf_2_)] (**10**)	1.0	2	56
2	[Au(IPent)(NTf_2_)] (**7**)	1.0	2	38
3	[Au(IHept)(NTf_2_)] (**8**)	1.0	2	13
4	[Au(INon)(NTf_2_)] (**9**)	1.0	2	16

^a^Reaction conditions: [Au(NHC)(NTf_2_)] (1.0 mol %), 4-methoxybenzonitrile (0.5 mmol), THF (0.5 mL), water (0.5 mL). ^b1^H NMR conversion, average of at least two runs.

#### Synthesis of homoallylic ketones

After the observed trend in hydration transformations, we focused our attention on non-water inclusive reactions and decided to explore the catalytic activity of complexes **7**–**9** in the synthesis of homoallylic ketones via hydroalkoxylation/Claisen rearrangement [61–62]. [Au(NHC)(NTf_2_)] complexes have proven to be efficient catalysts for this transformation, promoting the reaction under neat conditions and low catalyst loadings [61].

The reactions were conducted under the reported optimised conditions [61] and were analysed after 20 min. An inverse trend was observed in this case: the increase in the length of the alkyl chain resulted in lower conversions (Table 3). Complex **10** (Table 3, entry 1) was also found to be more efficient than complexes **7**–**9** for this transformation (Table 3, entries 2–4). Complex **7**, containing the IPent ligand, was the most active catalyst among the ITent series (Table 3, entry 2).

**Table 3:** Influence of the ITent ligands in the gold-catalysed synthesis of homoallylic ketones.^a^

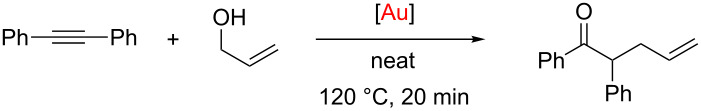			

entry	complex	[Au] (mol %)	conversion (%)^b^

1	[Au(IPr)(NTf_2_)] (**10**)	0.2	98
2	[Au(IPent)(NTf_2_)] (**7**)	0.2	81
3	[Au(IHept)(NTf_2_)] (**8**)	0.2	73
4	[Au(INon)(NTf_2_)] (**9**)	0.2	56

^a^Reaction conditions: [Au(NHC)(NTf_2_)] (0.2 mol %), diphenylacetylene (1.0 mmol), allylic alcohol (3 equiv). ^b^GC conversion, average of 3 runs.

## Conclusion

In conclusion, three new gold complexes bearing the ITent ligands (IPent, IHept, and INon) have been synthesised and fully characterised. The impact of varying the length of the alkyl chain of the ligands in gold-promoted transformations has been explored. All gold complexes were shown to be active in water inclusive reactions (alkyne and nitrile hydration) and in the synthesis of homoallylic ketones from allylic alcohols and alkynes. [Au(IHept)(NTf_2_)] was the most efficient complex of the series in the hydration of alkynes while the [Au(IPent)(NTf_2_)] analogue was found to be superior in the hydration of nitriles and in the synthesis of homoallylic ketones. However, when the performance of the catalysts was compared to that of the parent [Au(IPr)(NTf_2_)] complex, this appeared to be more active than the remaining complexes, showing that an increase of the alkyl chain length of the ligands has a detrimental effect in the gold-mediated transformations selected in this study. Further studies on the catalytic activity of the ITent ligands with different metals are currently ongoing.

## Supporting Information

File 1 Experimental information and full characterisation of the complexes including a copy of the NMR spectra.
