# Supplementary material for: Influence of bulky yet flexible N-heterocyclic carbene ligands in gold catalysis
Source: Beilstein J Org Chem. 2015 Oct 2;11:1809–14. doi: 10.3762/bjoc.11.196 (PMC4661005; doi:10.3762/bjoc.11.196)
Supplement: File 1 — Experimental information and full characterisation of the complexes including a copy of the NMR spectra. [file Beilstein_J_Org_Chem-11-1809-s001.pdf]

Supporting Information

for

Influence of bulky yet flexible *N*-heterocyclic carbene ligands in gold catalysis

Alba Collado[1], Scott R. Patrick[1], Danila Gasperini[1], Sebastien Meiries[1] and Steven P. Nolan[2*]

Address: [1]EaStCHEM School of Chemistry, University of St Andrews, St Andrews, KY16 9ST (UK) and [2]Chemistry Department, College of Science, King Saud University, Riyadh 11451, Saudi Arabia

Email: Steven P. Nolan[*] - stevenpnolan@gmail.com

* Corresponding author

Experimental information and full characterisation of the complexes including a copy of the NMR spectra

Table of contents

- General information……………………………………………………S2
- Synthesis of the gold complexes…………………………………S2
- Catalytic experiments…………………………………………………S5
- Copy of NMR spectra for all compounds……………………S6
- References……………………………………………………………………S10

General information

- All the reactions were conducted under air and technical grade solvents were used.
- ITent·HCl salts were prepared following the reported procedure.[1] [Au(ITent)(OH)] complexes were prepared following the reported procedure.[2] [Au(IPr)(NTf_2)] was prepared following a literature procedure.[3] $HNTf_2$ was obtained from Sigma Aldrich.
- Other reagents were obtained from commercial sources and used without further purification.
- Deuterated $CDCl_3$ was purchased from Acros and dried over molecular sieves.
- ^1H, $^{13}C\{^1H\}$, and $^{19}F\{^1H\}$ Nuclear Magnetic Resonance (NMR) spectra were recorded on a Bruker-400 MHz or 300 MHz spectrometer at ambient temperature in $CDCl_3$. Chemical shifts (expressed in parts per million) are referenced to residual solvent peaks.
- Elemental analyses were performed at London Metropolitan University 166-220 Holloway Road, London, N7 8DB.

Synthesis of the gold complexes

Preparation of [Au(IPent)(NTf_2)] (7): A vial was charged, under air, with [Au(IPent)(OH)] (50.0 mg, 0.070 mmol) and $HNTf_2$ (21.6 mg, 0.077 mmol). The resulting mixture was dissolved in toluene (1.0 mL) and stirred for 1 h at room temperature. After this time the mixture was filtered through Celite® and rinsed with toluene (3 × 0.5 mL). The solvent was concentrated and pentane (2.0 mL) was added, affording a white solid which was washed with further portions of pentane (3 × 1.0 mL) and dried under vacuum. Yield: 47.1 mg (68%).

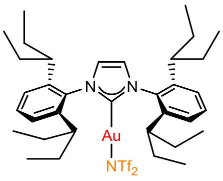

^{1}H NMR (300 MHz, $CDCl_3$) δ 7.52 (t, J = 7.8 Hz, 2H, CH_{Ar}), 7.23 (d, J = 7.8 Hz, 4H, CH_{Ar}), 7.14 (s, 2H, $CH^{4,5}$), 2.13-2.04 (m, 4H, CH), 1.87-1.60 (m, 12H, CH_2), 1.56-1.43 (m, 4H, CH_2), 0.91 (t, J = 7.4 Hz, 12H, CH_3), 0.78 (t, J = 7.4 Hz, 12H, CH_3). ^{13}C{^{1}H} NMR (75 MHz, $CDCl_3$) δ 167.8 (C_{carb}), 143.3 (C_{Ar}), 136.2 (C_{Ar}), 130.5 (CH_{Ar}), 124.9 (CH_{Ar}), 124.3 ($CH^{4,5}$), 119.1 (q, $J_{C\text{-}F}$ = 323.5, CF_3), 43.0 (CH), 29.1, and 28.0 (CH_2), 12.6 and 12.5 (CH_3). ^{19}F{^{1}H} NMR (376 MHz, $CDCl_3$). δ -76.08 (s, CF_3). Anal. Calcd. for $C_{37}H_{52}AuF_6N_3O_4S_2$: C 45.44; H 5.36; N 4.30. Found: C 45.55; H 5.43; N 4.40.

Preparation of [Au(IHept)(NTf_2)] (8): A vial was charged, under air, with [Au(IHept)(OH)] (50.0 mg, 0.060 mmol) and $HNTf_2$ (18.7 mg, 0.067 mmol). The resulting mixture was dissolved in toluene (1.0 mL) and stirred for 1 h at room temperature. After this time the mixture was filtered through Celite® and rinsed with toluene (3 × 0.5 mL). The solvent was concentrated and pentane (2.0 mL) was added, affording a white solid which was washed with further portions of pentane (3 × 1.0 mL) and dried under vacuum. Yield: 63.7 mg (97%).

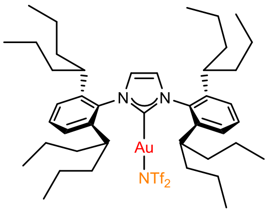

^{1}H NMR (300 MHz, $CDCl_3$) δ 7.50 (t, J = 7.8 Hz, 2H, CH_{Ar}), 7.23 (d, J = 7.8 Hz, 4H, CH_{Ar}), 7.13 (s, 2H, $CH^{4,5}$), 2.19 (p, J = 7.8 Hz, 4H, CH), 1.76-1.66 (m, 4H, CH_2), 1.65-1.53 (m, 8H, CH_2), 1.49-1.35 (m, 8H, CH_2), 1.25-1.13 (m, 8H, CH_2), 1.12-1.02 (m, 4H, CH_2), 0.87 (t, J =

7.3 Hz, 12H, CH_3), 0.83 (t, J = 7.3 Hz, 12H, CH_3). $^{13}C\{^1H\}$ NMR (75 MHz, $CDCl_3$) δ 167.5 (C_{carb}), 144.1 (C_{Ar}), 135.5 (C_{Ar}), 130.5 (CH_{Ar}), 124.8 (CH_{Ar}), 124.4 ($CH^{4,5}$), 119.0 (q, J_{C-F} = 323.4, CF_3), 40.2 (CH), 39.9, 37.9, 21.9, and 21.3 (CH_2), 14.5 and 14.4 (CH_3). $^{19}F\{^1H\}$ NMR (376 MHz, $CDCl_3$). δ -76.04 (s, CF_3). Anal. Calcd. for $C_{45}H_{68}AuF_6N_3O_4S_2$: C 49.58; H 6.29; N 3.85. Found: C 49.72; H 6.39; N 3.91.

Preparation of [Au(INon)(NTf_2)] (9): A vial was charged, under air, with [Au(INon)(OH)] (50.0 mg, 0.042 mmol) and $HNTf_2$ (12.9 mg, 0.046 mmol). The resulting mixture was dissolved in toluene (1.0 mL) and stirred for 1 h at room temperature. After this time the mixture was filtered through Celite® and rinsed with toluene (3 × 0.5 mL). The solvent was concentrated and pentane (2.0 mL) was added, affording a white solid which was washed with further portions of pentane (3 × 1.0 mL) and dried under vacuum. Yield: 63.5 mg (99%).

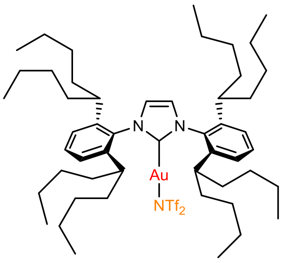

^1H NMR (300 MHz, $CDCl_3$) δ 7.51 (t, J = 7.8 Hz, 2H, CH_{Ar}), 7.24 (d, J = 7.8 Hz, 4H, CH_{Ar}), 7.13 (s, 2H, $CH^{4,5}$), 2.20 (p, J = 7.0 Hz, 4H, CH), 1.78-1.55 (m, 12H, CH_2), 1.48-1.11 (m, 32H, CH_2), 1.09-1.00 (m, 4H, CH_2), 0.86 (t, J = 7.1 Hz, 12H, CH_3), 0.85 (t, J = 7.0 Hz, 12H, CH_3). $^{13}C\{^1H\}$ NMR (75 MHz, $CDCl_3$) δ 167.7 (C_{carb}), 144.1 (C_{Ar}), 135.5 (C_{Ar}), 130.5 (CH_{Ar}), 124.8 (CH_{Ar}), 124.3 ($CH^{4,5}$), 119.0 (q, J_{C-F} = 323.3, CF_3), 40.2 (CH), 37.3, 35.0, 30.4, 30.2, 23.2 and 23.1 (CH_2), 14.2 and 13.9 (CH_3). $^{19}F\{^1H\}$ NMR (376 MHz, $CDCl_3$). δ -76.04 (s,

CF_3). Anal. Calcd. for $C_{53}H_{84}AuF_6N_3O_4S_2$: C 52.94; H 7.04; N 3.49. Found: C 53.02; H 7.17; N 3.58.

Catalytic experiments

Hydration of phenylacetylene. Under air, a vial was charged with phenylacetylene (0.5–1.0 mmol) and 1–2 mL of a 2:1 of dioxane/water solution. The corresponding amount of gold complex was added and the mixture was stirred at 80 °C for 1–3 h. After this time, pentane was added to the mixture to extract the organic product and the organic phase was analysed by GC analysis.

Hydration of 4-methoxybenzonitrile. Under air, a microwave vial was charged with 4-methoxybenzonitrile (66.6 mg, 0.50 mmol) and 1 mL of a 1:1 THF/water solution. The corresponding amount of gold complex was added and the mixture was stirred at 140 °C under microwave irradiation for 2 h. After this time, an aliquot of the mixture was analysed by ^1H NMR spectroscopy.

Hydroalkoxylation/Claisen rearrangement of alkynes. In a scintillation vial, diphenylacetylene (1 mmol), allylic alcohol (3 mmol) and [Au(NHC)(NTf_2)] (0.2 mol %), were stirred neat at 120 °C. After 20 min, the mixture was analysed by GC analysis.

NMR spectra

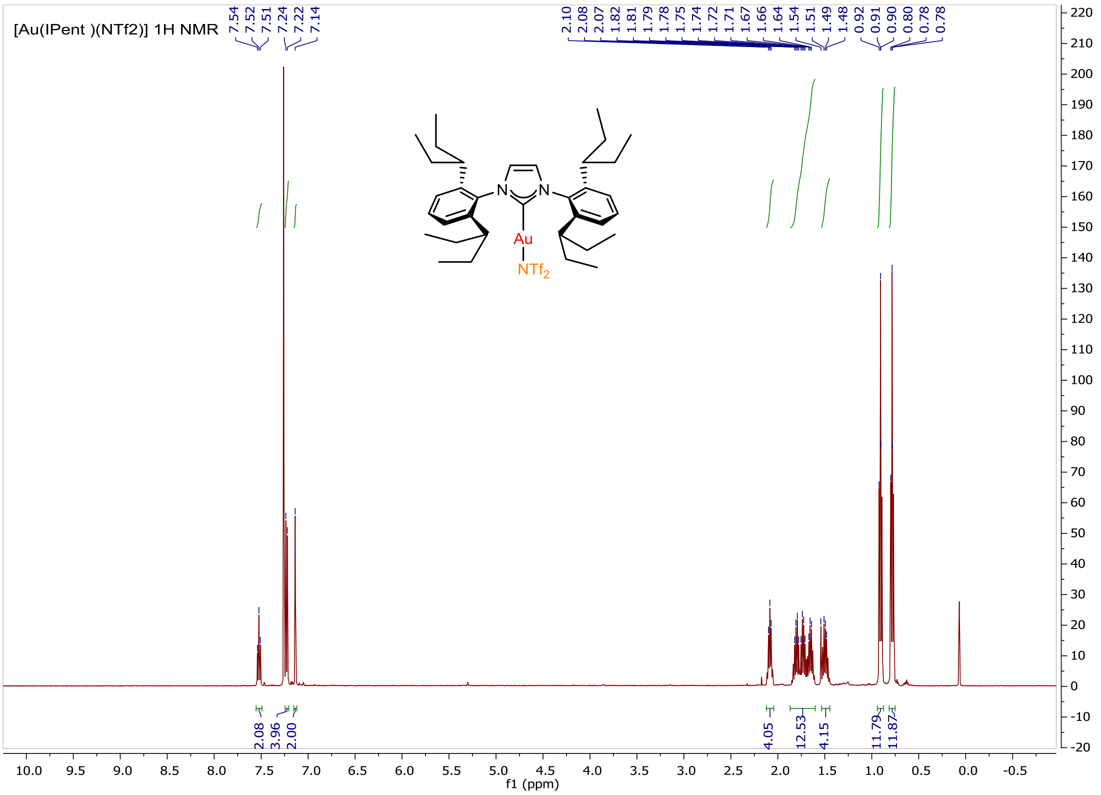
[Au(IPent)(NTf2)] 1H NMR
7.54
7.52
7.51
7.24
7.22
7.14
2.10
2.08
2.07
1.82
1.81
1.79
1.78
1.75
1.74
1.72
1.71
1.67
1.66
1.64
1.54
1.51
1.49
1.48
0.92
0.91
0.90
0.80
0.78
0.78
2.08
3.96
2.00
4.05
12.53
4.15
11.79
11.87
N
N
Au
NTf2
f1 (ppm)

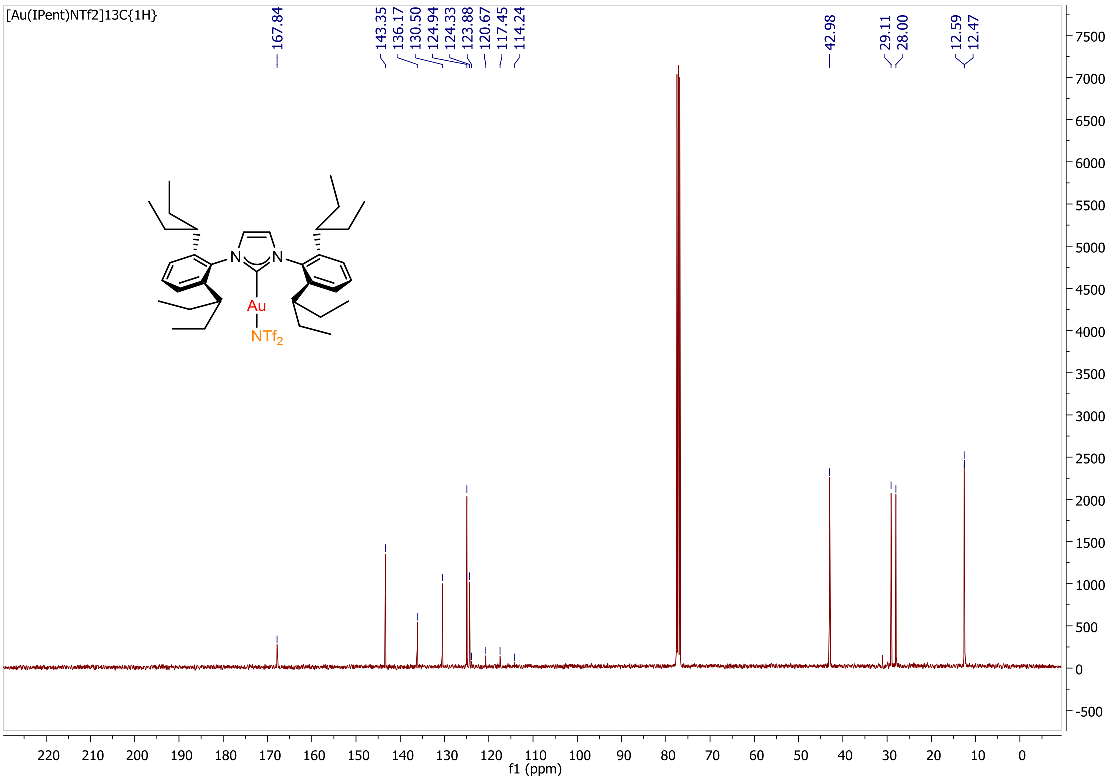
[Au(IPent)NTf2]13C{1H}
167.84
143.35
136.17
130.50
124.94
124.33
123.88
120.67
117.45
114.24
42.98
29.11
28.00
12.59
12.47
N
N
Au
NTf2
f1 (ppm)

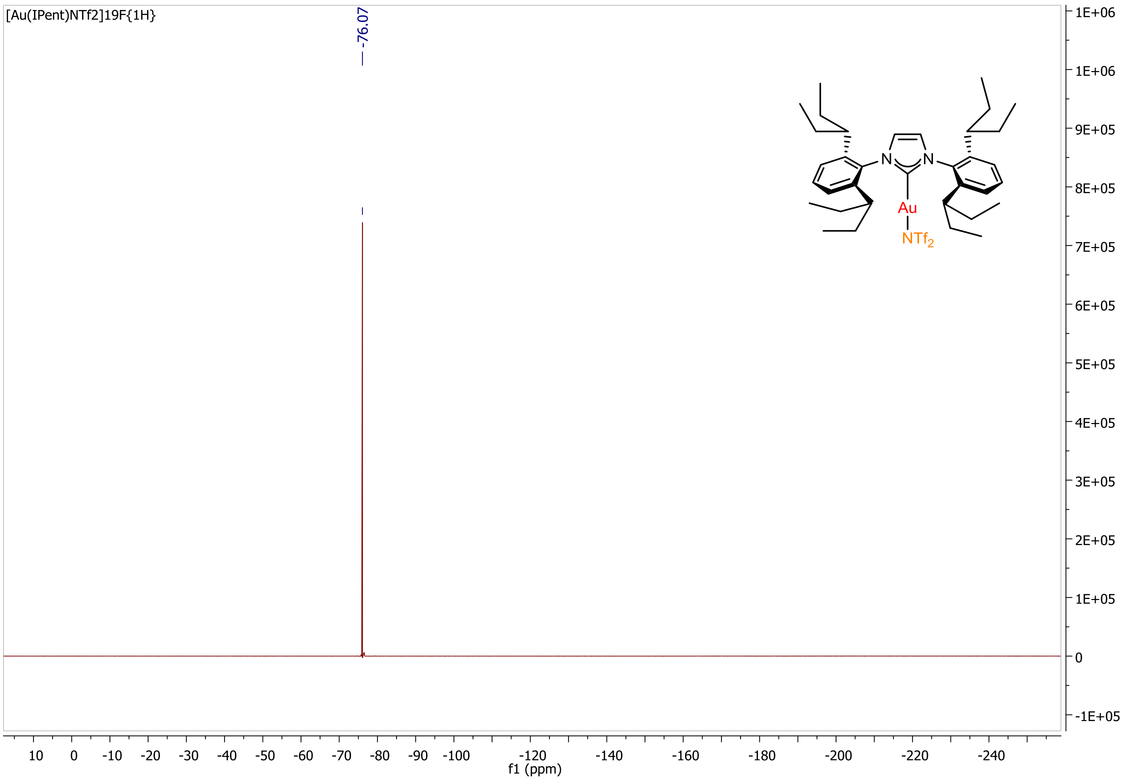
[Au(IPent)NTf2]19F{1H}
-76.07
Au
NTf2
f1 (ppm)

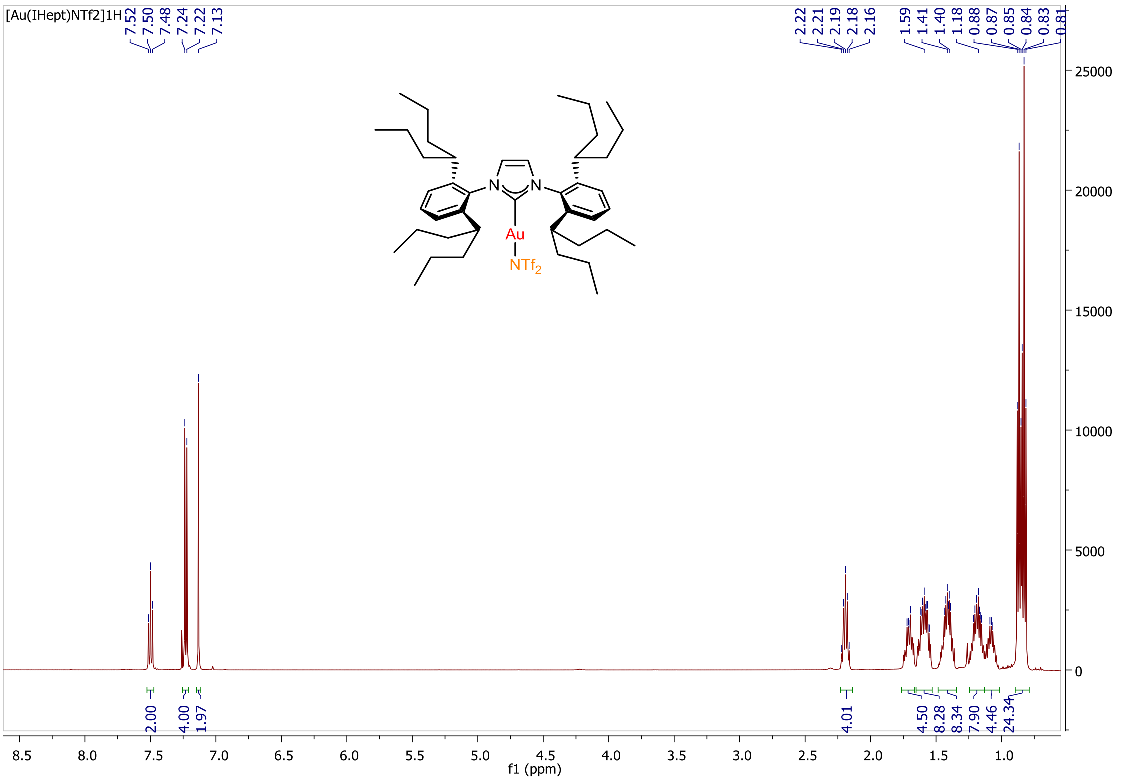
[Au(IHept)NTf2]1H
7.52
7.50
7.48
7.24
7.22
7.13
2.22
2.21
2.19
2.18
2.16
1.59
1.41
1.40
1.18
0.88
0.87
0.85
0.84
0.83
0.81
Au
NTf2
2.00
4.00
1.97
4.01
4.50
8.28
8.34
7.90
4.46
24.34
f1 (ppm)

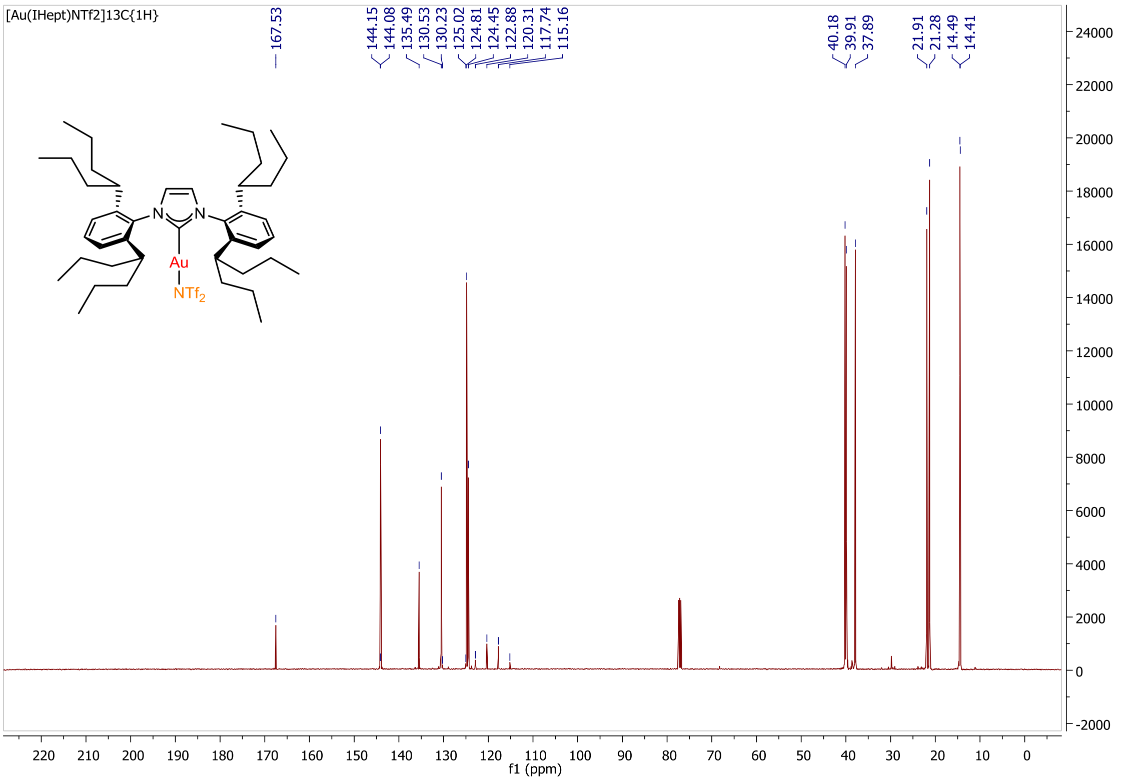
[Au(IHept)NTf2]13C{1H}
167.53
144.15
144.08
135.49
130.53
130.23
125.02
124.81
124.45
122.88
120.31
117.74
115.16
40.18
39.91
37.89
21.91
21.28
14.49
14.41
N
N
Au
NTf_2
f1 (ppm)

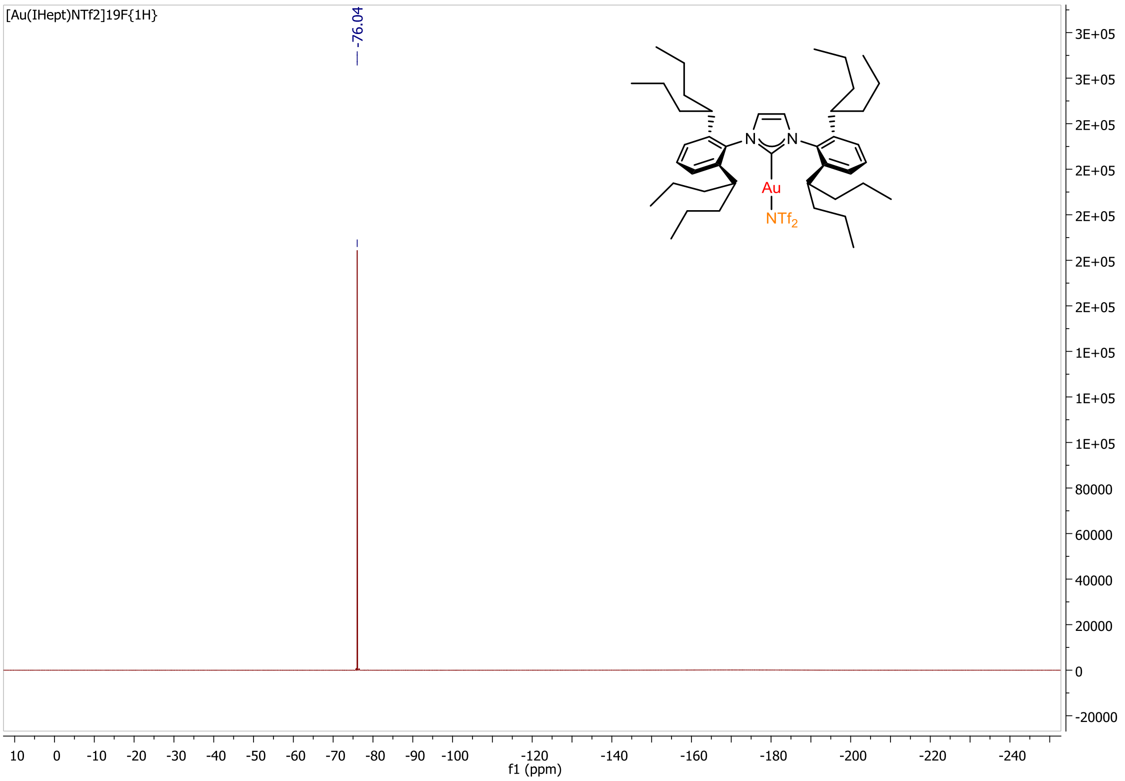
[Au(IHept)NTf2]19F{1H}
-76.04
N
N
Au
NTf_2
f1 (ppm)

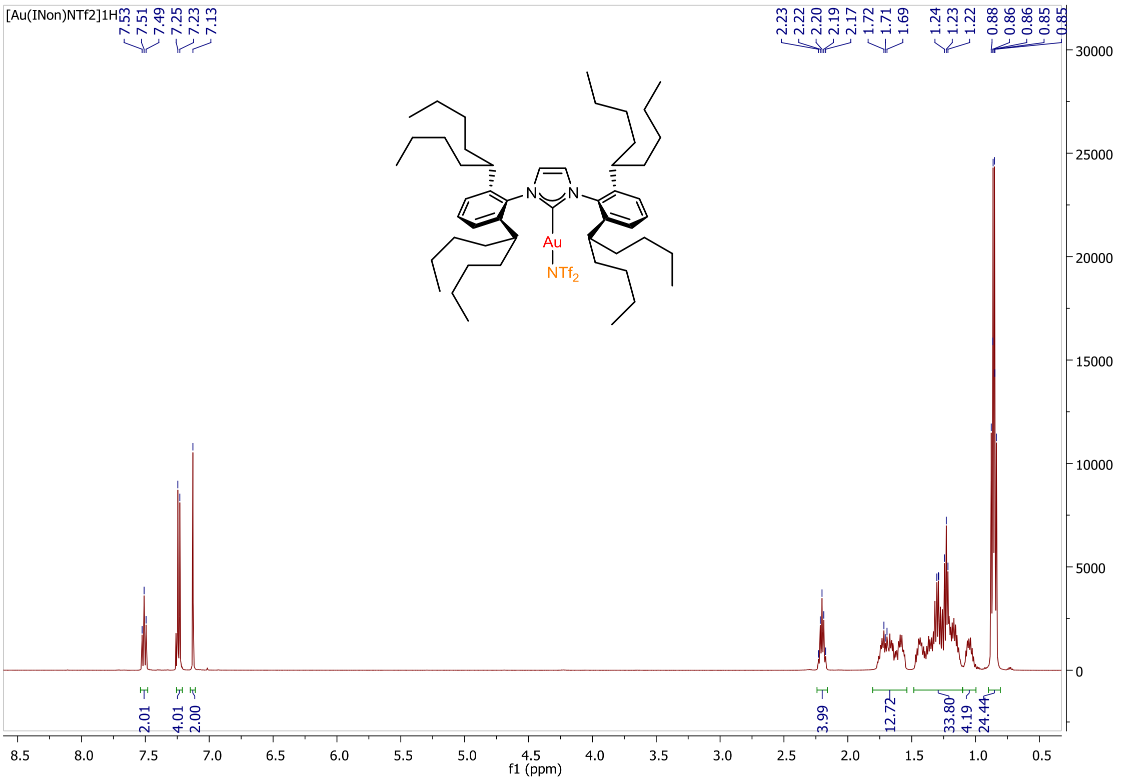
[Au(INon)NTf2]1H
7.53
7.51
7.49
7.25
7.23
7.13
2.23
2.22
2.20
2.19
2.17
1.72
1.71
1.69
1.24
1.23
1.22
0.88
0.86
0.86
0.85
0.85
2.01
4.01
2.00
3.99
12.72
33.80
4.19
24.44
f1 (ppm)
N
N
Au
NTf2

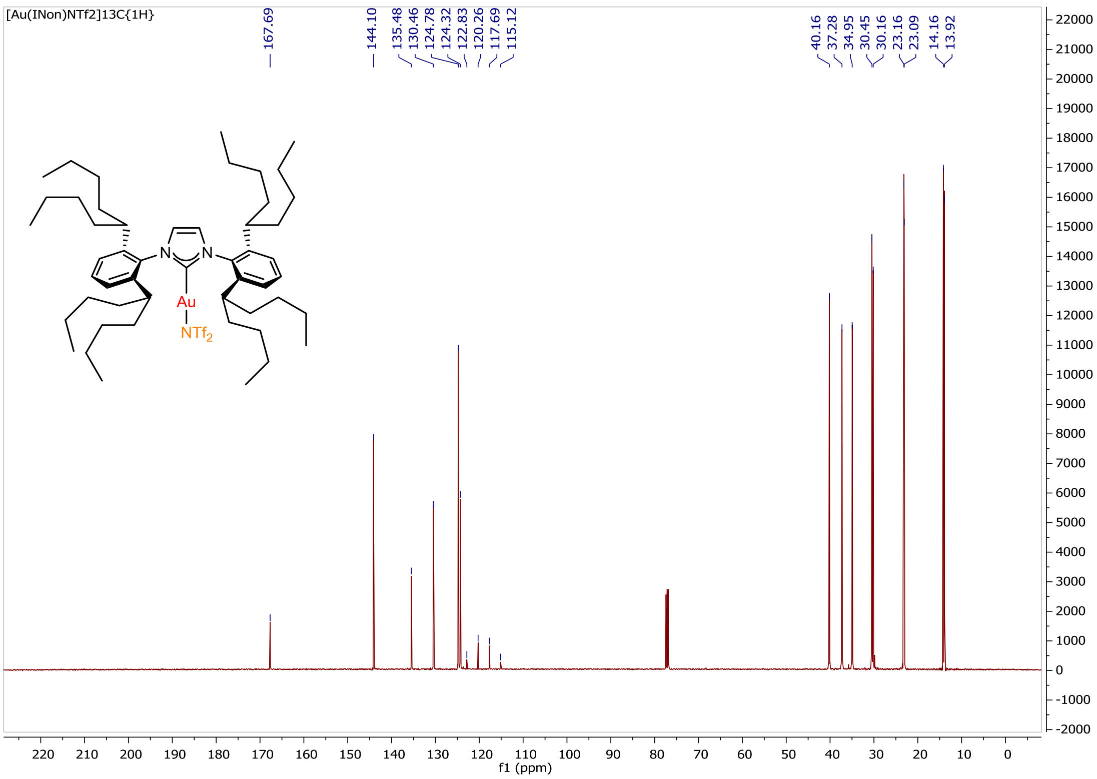
[Au(INon)NTf2]13C{1H}
167.69
144.10
135.48
130.46
124.78
124.32
122.83
120.26
117.69
115.12
40.16
37.28
34.95
30.45
30.16
23.16
23.09
14.16
13.92
f1 (ppm)
N
N
Au
NTf2

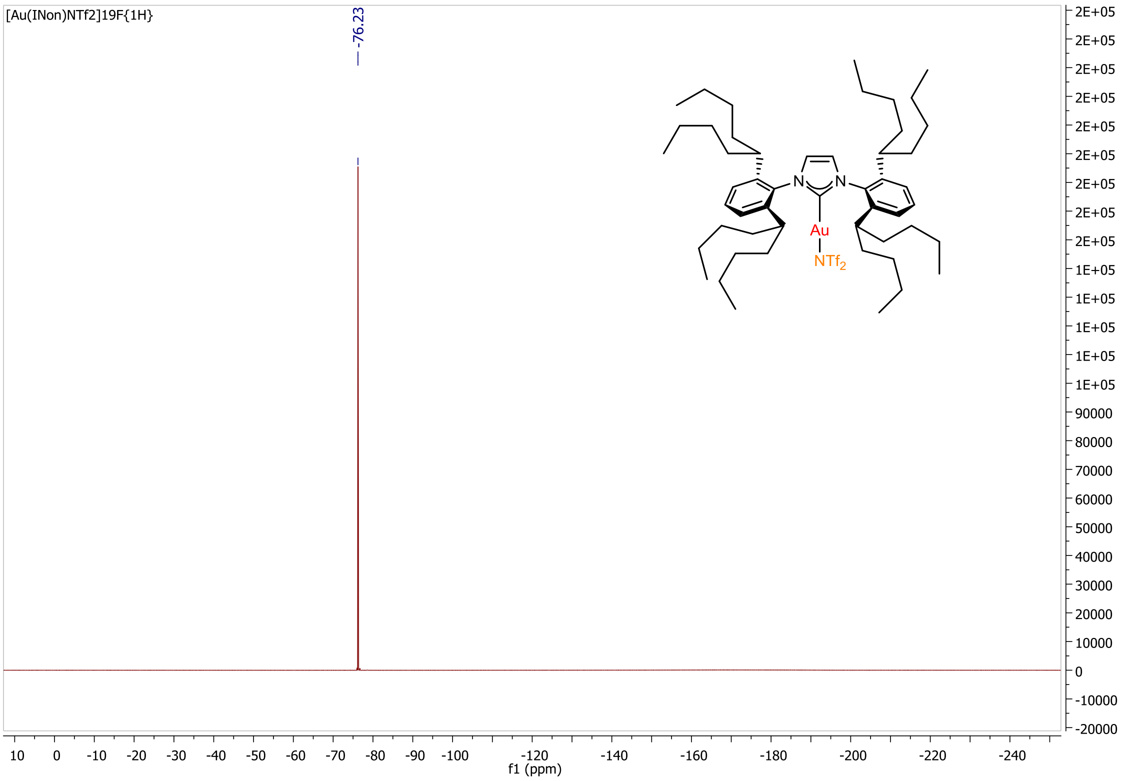

References

1. Meiries, S.; Le Duc, G.; Chartoire, A.; Collado, A.; Speck, K.; Arachchige, K. S. A.; Slawin, A. M. Z.; Nolan, S. P., *Chem. Eur. J.* **2013,** *19*, 17358.

2. Patrick, S. R.; Collado, A.; Meiries, S.; Slawin, A. M. Z.; Nolan, S. P., *J. Organomet. Chem.* **2015,** *775*, 152.

3. Gaillard, S.; Slawin, A. M. Z.; Nolan, S. P., *Chem. Commun.* **2010,** *46*, 2742.
